# The half plane UIPT is recurrent

**DOI:** 10.1007/s00440-017-0767-z

**Published:** 2017-03-07

**Authors:** Omer Angel, Gourab Ray

**Affiliations:** 1 Department of Mathematics, University of British Columbia, Vancouver, Canada; 2 Statistical Laboratory, University of Cambridge, Cambridge, UK

**Keywords:** 60B99, 60G50

## Abstract

We prove that the half plane version of the uniform infinite planar triangulation (UIPT) is recurrent. The key ingredients of the proof are a construction of a new full plane extension of the half plane UIPT, based on a natural decomposition of the half plane UIPT into independent layers, and an extension of previous methods for proving recurrence of weak local limits (while still using circle packings).

## Introduction

The half plane uniform infinite planar triangulation, abbreviated as the HUIPT below, is a random planar triangulation, closely related to the well-known and extensively studied uniform infinite planar triangulations (UIPT), but with the topology of the half plane. The HUIPT is an interesting object in its own right, and in some ways is nicer than the UIPT. For example, it possesses a simpler form of the domain Markov property (defined in Sect. 2). The problem of establishing the recurrence of the UIPT had been open for many years. This problem was a motivation for the seminal work of Benjamini and Schramm [14], and was resolved in recent work of Gurel-Gurevich and Nachmias [20]. However, recurrence of the HUIPT does not follow from their work, as it is not known if it is possible to realize the HUIPT as a subgraph of the UIPT. Indeed, there are some indications that such a coupling does not exist. For example, recent works of Caraceni and Curien [15] and of Gwynne and Miller [21] show that gluing two copies of the HUIPQ along their boundary results in a map with law singular relative to the UIPQ. Once the UIHPT is shown to have the same scaling limit as the UIHPQ, the singularity result will hold for triangulations as well. In this article we establish the recurrence of the half-plane UIPT.

### Theorem 1

The simple random walk on the half plane uniform infinite planar triangulation is almost-surely recurrent.

While our proof incorporates some ideas from [14, 20], new methods are also needed. A crucial ingredient in those works is that the graphs under consideration are weak local limits of finite planar graphs, with a root that is chosen uniformly among all vertices. After embedding the graphs in a carefully chosen manner in the plane, this leads to a fundamental Lemma on the geometry of arbitrary point sets in the plane [14, Lemma 4.2]. A quantitative version of this lemma [20, Lemma 3.4] was exploited to prove the recurrence of the UIPT, and is used in this work as well (see Lemma 6.1 below).

Crucially, the methods of [14, 20] do not apply directly, since the HUIPT is not a weak local limit of finite planar graphs. The earliest construction of the HUIPT is as a weak limit of uniform triangulations with boundaries where the root is restricted to the boundary. In particular, the root is not a uniform vertex. The main novelty of this work lies in the technique used to overcome this obstacle. Along the way we obtain a certain random full plane map *M* which we call the layered UIPT. The layered UIPT contains the HUIPT as a subgraph. We believe *M* to be of independent interest and to have further applications. We prove that *M* is recurrent which implies that the HUIPT is recurrent.

Another difficulty stems from the fact that (unlike the UIPT), the HUIPT is not stationary for the simple random walk. Indeed, viewed from the random walker, the HUIPT should converge in distribution w.r.t. the local topology to the UIPT, as the walker will typically be far from the boundary. The map *M* we introduce is not stationary itself, but there is a certain local modification of *M* which is stationary, and even reversible. Thus in a certain sense, the map *M* can be seen as a stationary reversible version of the HUIPT. (A random rooted graph $(G,\rho )$ is stationary reversible with respect to simple random walk $\{\rho ,X_1,\dots \}$ if the law of the doubly marked graph $(G,\rho ,X_1)$ is the same as the law of $(G,X_1,\rho )$ see [10]; reversibility and the related property of unimodularity has been exploited in the past to great advantage in a number of settings [5–7, 10–13].)

Finally, a central tool we use is a decomposition of the HUIPT and of the layered UIPT into independent layers (see Sect. 3). An analogous decomposition was used by Krikun [25, 26] for the UIPQ. However, the domain Markov property of HUIPT gives this decomposition a particularly elegant structure. Such a decomposition has great potential for the study of random maps. A recent work of Curien and Le Gall [19] analyzes first passage percolation and other perturbations of the metric structure of the UIPT via such a decomposition. A continuum version of this decomposition has been introduces in recent work of Miller and Sheffield as part of a characterization of the Brownian map [28].

### Outline of proof

A naive approach to proving recurrence of the HUIPT is to use the result of Gurel-Gurevich and Nachmias in [20]. Let $B_r$ be the hull of the combinatorial ball of radius *r* around the root (the hull is obtained by adding the finite components of the complement of the ball). If a root $\rho _r$ is chosen uniformly from *all the vertices* in $B_r$, then the resulting sequence of rooted finite planar graphs $(B_r,\rho _r)$ has an exponential tail on the degrees, and thus their limit would be recurrent almost surely. If the root is near the boundary, then the limit is the HUIPT. However, the root is unlikely to be near the boundary, and the limit above is the full plane UIPT. If we can show that the limit contains *H* as a subgraph, then we would be done. However, as noted above, inclusion of the HUIPT in the UIPT is an open problem.

A more refined approach is to find some subset *S* of the vertices of the ball such that if we pick uniformly a root uniformly from *S* we obtain a limit which contains *H* as a subgraph. One natural choice is to set *S* to be $\partial B_r$, so that the limit is the HUIPT. However, since $|\partial B_r| \approx r^2$, this set is much smaller than the volume of $B_r$. Thus the limit is not absolutely continuous with respect to the weak local limit of $B_r$, and we are still short of a proof.

An improvement would be to take *S* to be the union of the boundaries $\partial B_j$, for $1 \le j \le r$. This set is still much smaller than the volume of $B_r$. However, the situation can be salvaged: This set *S* disconnects the balls into small components (the blocks below); Understanding the structure of *S* gives some control over the structure of the resulting limit. One can circle pack the limiting graph, and the circles corresponding to the set *S* will have no accumulation points in the plane. Moreover, Lemma 6.1 gives us control over the number of vertices of *S* in a Euclidean ball. In practice, it is more convenient to replace $B_r$ by a different subgraph of the HUIPT, which is done below in Sect. 4.

In order to complete the proof, we also need some new estimates on the volume of balls in the HUIPT under a certain modified metric, as well as estimates on vertex degrees. With these in place, we can push through the proof of [20].

We comment that there are other natural measures on half planar maps. This includes the half plane quadrangulation, or half plane map with unrestricted face sizes, simple triangulations, and more. There seems to be no crucial obstacle to extending our results to such several other classes of maps. We restrict our focus in this paper to the case of triangulations where the the layer decomposition is particularly nice. As noted, a similar decomposition was used by Krikun for quadrangulations, and with some care it seems the layered structure as well as the rest of our argument can be adapted to the half plane quadrangulation.

*Organization*

In Sect. 2 we include some background material which we use, concerning the weak local topology, planar maps, the UIPT and HUIPT, and circle packings. Readers familiar with these topics may wish to skip to Sect. 3 where we describe the layer decomposition of the HUIPT, and describe the full plane map *M* containing the HUIPT. We also prove there estimates on the volume growth and vertex degrees in *M*. In Sect. 4 we show that a certain sequence of finite maps with suitable distribution for the root converge to *M*. Finally, in Sect. 6 we combine all ingredients and prove Theorem 1. We end with some comments on possible extensions and open questions in Sect. 7.

## Background

### Planar maps: the UIPT and relatives

Recall that a planar map is a proper embedding in the plane of a connected (multi) graph in the plane, considered up to orientation preserving homeomorphisms. Components of the complement of the map are called faces, and are assumed to be simple discs. All our maps are rooted, meaning there is a marked directed edge, called the root. Equivalently, a planar map is a graph together with a cyclic order on the edges at each vertex, such that the graph can be embedded with the edges leaving the vertex in order.

Our maps will have a distinguished face which we shall call the external face. The edges and vertices incident to the external face will be called the boundary of the map. When a map has a boundary, we shall often assume the root is one of the boundary edges. The boundary throughout this paper will be either a simple cycle or a simple doubly infinite path (a sequence of directed edges $\{e_i\}_{i \in {\mathbb {Z}}}$ such that the tail of $e_i$ is the head of $e_{i+1}$). In the latter case, the map may be embedded in the half plane with the boundary along a line. Such a map is referred to as a half plane map. A half plane map where all the faces except the boundary face are triangles is called a half planar triangulation.

The local topology on the space of rooted graphs is generated by the following metric: for rooted graphs *G*, *H*, we define

$$\begin{aligned} d(G,H) = e^{-R} \qquad \text {where} \qquad R = \sup \{r: B_r(G) \cong B_r(H)\}. \end{aligned}$$

Here $B_r$ denotes the ball of radius *r* around the corresponding roots in the graph distance, and $\cong$ denotes isomorphism of rooted maps. For maps, we require the equivalence relation to preserve the cyclic order on edges at vertices.

This topology on graphs or maps induces a weak topology on the space of measures on graphs (resp. maps). A finite, possibly random, graph yields a measure on rooted graphs by taking the root to be a uniform directed edge (or vertex). The weak local limit (or Benjamini–Schramm limit) of a sequence of finite graphs is the weak limit of the induced measures. The starting point of our work is the following result of Gurel-Gurevich and Nachmias (and of Benjamini and Schramm with a bounded degree assumption).

#### Theorem 2.1

[14, 20] Let $G_n$ be finite planar graphs such that the degree of a uniform vertex has uniformly exponential tail. Then $\lim G_n$ is almost surely recurrent.

It has been known for some time [3, 8, 9] that the uniform measures on finite planar triangulations with boundary converge in the weak local topology as the area of the map and the boundary length tend to infinity.

Let ${\mathcal {T}}_n$ denote the set of all rooted triangulations with no boundary, with *n* faces. Let ${\mathcal {T}}_{n,m}$ denote the set of all rooted triangulations with a boundary (meaning all faces are triangles, except possibly the boundary face) with *m* boundary vertices and *n* non-boundary faces where the root is a boundary edge with the boundary face on its left.

#### Theorem 2.2

[8, 9] If $T_n$ is a triangulation chosen uniformly from ${\mathcal {T}}_{n}$, then the limit $T = \lim T_n$ exists (in the weak local topology). If $T_{n,m}$ is uniformly chosen from ${\mathcal {T}}_{n,m}$, then we have the limit

$$\begin{aligned} T_{n,m} \xrightarrow [m,n/m \rightarrow \infty ]{d} H. \end{aligned}$$

The limits *T* and *H* are the UIPT and half plane UIPT. We denote the law of *H* by ${\mathbb {H}}$.

The map *H* also enjoys translation invariance with respect to the root. This means that the law of the map remains invariant if we translate the root along the boundary. See [8] for a detailed definition.

The distribution of a neighbourhood of the root in the HUIPT has a simple and explicit formula which can be taken as an alternative direct definition of HUIPT.

#### Lemma 2.3

[8] Let *Q* be a finite simply connected triangulation with a simple boundary, with some marked connected segment of $\partial Q$ containing the root, and let *H* be the HUIPT. Consider the event $A_Q$ that *Q* is a sub-map of *H* with the roots coinciding and the marked segment being the intersection of *Q* with $\partial H$. Then

$$\begin{aligned} {\mathbb {H}}(A_Q) = 6^{\#V_i(Q)} 9^{-\#F(Q)} \end{aligned}$$

where $\#V_i(Q)$ is the number of vertices of *Q* not in $\partial H$ and $\#F(Q)$ is the number of faces of *Q*. Moreover, conditioned on $A_Q$, the complement $H{\setminus }Q$ also has law ${\mathbb {H}}$.

The final claim of this lemma is referred to as the domain Markov property of the HUIPT (see [8]).

### Peeling

One of the main tools we are going to use is known as peeling which was introduced by Watabiki [29] and given its present form by Angel [3]. This technique can be applied to more general class of maps, we focus primarily on HUIPT. The central idea is to explore (or “peel”) a map face by face. There can be many possible algorithms to do it, and generally an algorithm is chosen depending on the purpose. The domain Markov property in the HUIPT gives the peeling process a rather simple form. For further applications of this powerful tool see e.g. [4, 8, 11, 17, 27].

Consider the unique triangle incident to the root edge of the half plane UIPT *H*. One of the following two events must occur: With probability 2 / 3, the triangle can be incident to an internal vertex. Otherwise the triangle incident to the root edge is attached to a vertex on the boundary which is at a distance *i* to the left (resp. right) of the root edge along the boundary. Let $p_i$ be the probability of this event. Moreover, let $p_{i,k}$ be the event that the finite face enclosed by such a triangle has *k* vertices. Let $\phi _{k,i}$ denote the number of triangulations of an *i*-gon with *k* internal vertices. The following were derived in [3].

2.1 $$\begin{aligned} \begin{aligned} p_{i,k}&= \phi _{k,i+1} \left( \frac{1}{9}\right) ^i \left( \frac{2}{27} \right) ^k \\ p_i&= \sum _kp_{i,k}=\frac{2}{4^i} \frac{(2i-2)!}{(i-1)!(i+1)!} \sim \frac{1}{2\sqrt{\pi }}i^{-5/2} \end{aligned} \end{aligned}$$

The Boltzmann triangulation of an *m*-gon with weight $q\le \frac{2}{27}$, is the probability measure on that assigns weight $q^n / Z_m(q)$ to each rooted triangulation of the *m*-gon having *n* internal vertices, where

$$\begin{aligned} Z_m(q) = \sum _n \phi _{n,m} q^n. \end{aligned}$$

The partition function $Z_m$ can be computed explicitly, and is finite for $|q|\le 2/27$. When peeling a face, on the event that the face connects to a vertex at distance *i*, the resulting component with boundary $i+1$ is filled with a Boltzmann triangulation with weight 2 / 27.

Having revealed the triangle incident to the root edge and the finite component of its complement (if any), the unrevealed map is another half plane map having law ${\mathbb {H}}$ by the domain Markov property. This enables us to peel the HUIPT via a succession of i.i.d. peeling steps. Note that the probabilities $p_{i,k}$ do not depend on the edge we choose to peel, by translation invariance.

### Circle packings

As in some prior works [6, 14, 20], circle packings play a central role for us. We state here the two key results needed. We refer the reader to [24] and the above papers for further information.

A circle packing of a graph *G* is a collection of circles in the plane with disjoint interiors, one corresponding to each vertex, such that two circles are tangent if and only if the corresponding vertices are adjacent. The Kobe-Andreev-Thurston Circle Packing Theorem states that every finite planar graph has a circle packing. There are extensions to infinite planar triangulations, which we do not need at present.

In order to control the geometry of graphs in terms of circle packings, it is useful to control the ratio of radii of circles. This is done by the so called Ring Lemma, which states that in a circle packing of a triangulation, the ratio of radii of adjacent circles is bounded by some constant depending only on the maximal degree of the graphs (for non-boundary vertices).

## Layer decomposition

We now define the layer decomposition of a half planar map. The definition makes sense for general maps, and some of the properties below will also hold for other classes of maps satisfying the domain Markov property. However, the combinatorics of the decomposition are particularly nice for triangulations. Thus we define the decomposition in general, but state and prove some of its properties only for triangulations.

Given a half planar map *H*, we define its layer decomposition as follows. For each *i*, we will have a half plane map $H_i$. These will form a decreasing family of sub-maps of *H*, and each is a half plane map. The boundary of $H_i$ is denoted $S_i$, and is a doubly infinite simple path in *H*. The vertices in $S_i$ are called skeleton vertices.

**Fig. 1:**
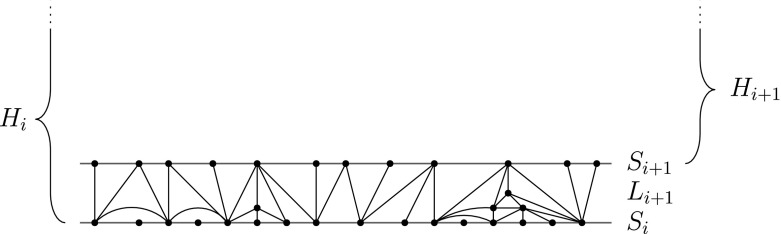
Construction of layer $L_i$ from $H_i$. $L_i$ is the hull of all faces incident to the boundary $S_i$. The entire HUIPT is $H_0$

Inductively, we start with $H_0=H$ and its boundary $S_0=\partial H_0$. Having defined $H_i$ and $S_i$, we define the layer $L_{i+1}$ as a sub-map of $H_i$. The layer is defined in terms of the set of its faces, and it also contains all edges and vertices of these faces. To define $L_{i+1}$, we start with the set *A* of faces of $H_i$ incident to the boundary of $H_i$, and take its hull where the hull is defined as follows. Let $H_i^*$ denote the dual map of $H_i$ (excluding the vertex for the external face). Let $A^*$ denote the graph induced by the dual vertices which correspond to the faces of *A*. Then the hull of *A* consists of *A* together with the dual faces corresponding to all finite components of $H_i^*{\setminus }A^*$. See Fig. 1.

Next, we define the next sub-map $H_{i+1} = H_i{\setminus }L_{i+1}$. Formally, this is a map containing all faces of $H_i$ not in $L_{i+1}$ (i.e. faces corresponding to the infinite component of $H_i^*\setminus A^*$), and all edges and vertices included in such faces. We set $S_{i+1}$ to be the boundary of $H_{i+1}$.

If no assumption is made about the map *H*, then it is possible that some layer $L_{i+1}$ is the entire map $H_i$, so that $H_{i+1}$ is empty. This happens for example for the sub-critical domain Markov maps defined in [8]. We will show below that if *H* is the HUIPT this does not occur, and hence for each *i* we have that $S_i$ is a simple doubly infinite path, see Lemma 3.1. This path is the intersection of the consecutive layers $L_{i}$ and $L_{i+1}$. Conversely, the boundary of $L_i$ is $S_i \cup S_{i+1}$. Note that by construction the sets $S_i$ are disjoint.

The attentive reader will note that we have not specified a root for the maps $H_i$. A root can be chosen for each $H_i$ in various manners, and we describe a specific one below. However, the construction of the sequence of layers and sub-maps is independent of the choice of root.

**Fig. 2:**
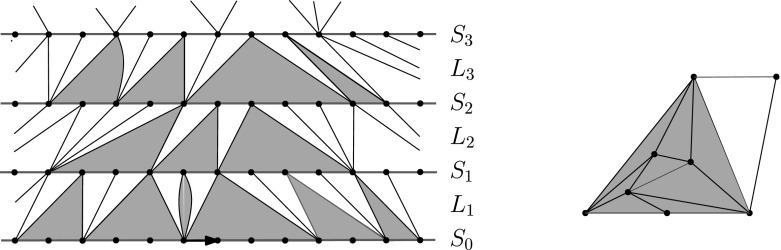
Left: Decomposition of layers into alternating holes and faces adjacent to top boundary edges. *Holes* are *shaded*, and *vertices* and *edges* within holes are not shown. Note that some holes degenerate. Right: A hole and face to its right form a block

From now on, we assume that *H* is a half planar triangulation with $S_i \ne \emptyset$ for all *i* unless otherwise specified. Let *e* be some edge in $S_{i+1}$ for some $i\ge 0$. Then there is a unique face in $L_{i+1}$ containing *e*. The third vertex of that face must be in $S_i$, since otherwise that face would not have been included in $L_i$. This triangle separates $L_i$ into a part on its left and a part on its right, with only the one vertex of $S_i$ in common. For two adjacent edges $e_1,e_2\in S_{i+1}$ (with $e_1$ on the left), the corresponding triangles, say $f_1$ and $f_2$, of $L_i$ that contain $e_1$ and $e_2$ split $L_i$ into two infinite and a finite (possibly empty) component, to the right of $f_1$ and left of $f_2$. We refer to the finite components arising in this way as holes. Note that it is possible that $f_1$ and $f_2$ share a common edge, in which case the hole degenerates to that single edge. It is also possible that $f_1,f_2$ share a vertex in $S_i$, but not a common edge. In that case the hole is a 2-gon. Both of these occur in the lowest layer in Fig. 2. This observation implies that $L_i$ can be decomposed as an alternating sequence of (possibly degenerate) holes and faces containing the edges of $S_{i}$. We can thus partition $L_i$ to a sequence of blocks, where each block consists of a hole and the triangle immediately to its right. The lower boundary of a block in $L_i$ is the set of edges of $S_{i-1}$ in the block, which can be any non-negative integer. (The upper boundary always consists of a single edge.) Apart from the lower and the upper boundary, the block has two more boundary edges, where it is attached to blocks to its left and right.

### Decomposition of the half plane UIPT

Up to this point we described the layer decomposition of a general half plane map. We now focus our attention on the specific case of the half-plane UIPT. While in our case, the description above is faithful, in arbitrary half-plane maps things could break down. In particular, it is possible that $L_1$ is the entire map. Indeed, this is the case in the sub-critical half plane maps with the domain Markov property that were constructed in [8].

#### Lemma 3.1

For the HUIPT, almost surely, $L_1$ is not the entire half plane, $S_1$ is a simple doubly infinite path and $H_1$ is also a half plane map. Moreover, if we choose a root for each $H_{i+1}$ as a function of $L_1,\dots ,L_i$, then $H_{i+1}$ has the law of the half plane UIPT, and is independent of $\{L_1,L_2,\ldots , L_i\}$. Consequently, the layers $\{L_i\}_{i \ge 1}$ are i.i.d.

*Peeling to reveal a layer*

To prove Lemma 3.1, it shall be useful to consider the following application of peeling in the half plane UIPT. An analogue of this for the UIPT was used in [3] to study the volume growth of the UIPT. In the HUIPT, the process becomes simpler. Initially, make the root edge active. At any later time, the active edges are those at the boundary of the unseen part of the map that are not on the original boundary. The active edges form a single contiguous segment, and we peel either the rightmost or leftmost active edge. Let $Y_n$ be the number of active edges in this segment after *n* steps (with $Y_0=1$).

Let $Y_n$ be the length of this segment after *n* steps, except that by convention we set $Y_0=1$. Define now the i.i.d. sequence $\xi _n$ as follows. If the *n*th step connects the peeled edge to a new internal vertex, then $\xi _n=1$. If it connects to a vertex at distance *i* towards the rest of the active segment then $\xi _n=-i$. Finally, if the face connects to a vertex to the right when we are peeling at the rightmost active edge, or to the left when we are peeling at the leftmost acrive edge, we have $\xi _n=0$. It is easy to see that $\xi _n$ determines the change in $Y_n$. Specifically, he have

3.1 $$\begin{aligned} Y_n = (Y_{n-1} + \xi _n) \vee 1. \end{aligned}$$

The $\xi _n$ variables are i.i.d. with distribution

3.2 $$\begin{aligned} {\mathbb {P}}(\xi =i) = {\left\{ \begin{array}{ll} 2/3 &{} i=1, \\ 1/6 &{} i=0, \\ p_i/2 &{} i<0, \end{array}\right. } \end{aligned}$$

where $p_i$ is given in (2.1). It follows from the computations in [3] that ${\mathbb {E}}(\xi ) = 1/3$. Note also that every peeled face is incident to some vertex in the original boundary, and so all faces revealed in this procedure are part of $L_0$. Finally, the number of edges of the original boundary that are swallowed at each step are also i.i.d. with mean 1 / 3. Note that some of the edges which were active before a peeling step might not remain active after the step. In effect, the active edges swallowed by the face revealed in the peeling step are removed from the active edge list.

#### Proof of Lemma 3.1

When peeling to reveal a layer, since ${\mathbb {E}}\xi =1/3>0$, the strong law of large numbers implies that $Y_n/n$ converges to 1 / 3 almost surely and in particular, $Y_n$ tends to infinity almost surely.

Start by peeling at the rightmost active edge *n* times. The law of large numbers ensures that the number of edges to the right of the root that are swallowed grows like *n* / 3. While some of the previously active edges contributing to $Y_n$ are swallowed at a later step, at each *n* there is probability 1 / 3 that $Y_n = \inf _{t\ge n} Y_t$ (via Theorem 3 of [1]), and in that case, only the rightmost active edge is subsequently swallowed. In particular, the number of boundary vertices to the left of the root that are swallowed is tight.

Next, reverse direction, and peel towards the left for *n* additional steps. At this time we revealed some finite map $P_n$ which contains all faces incident to edges within distance $a_n$ along the boundary to the left and $b_n$ along the boundary to the right. By the law of large numbers, both $a_n,b_n$ are close to *n* / 3 with high probability. Thus $P_n$ are an exhaustion of $L_1$, in that every face of $L_1$ is eventually in $P_n$. Moreover, the number of edges contributing to $Y_n$ that are swallowed in the second stage is tight, and therefore with high probability some of them remain on the boundary as $n\rightarrow \infty$. This implies that $L_1$ is not the entire map.

To see that $H_1$ is again a half plane UIPT, and is independent of $L_1$, root $M_n = H\setminus P_n$ at some canonically chosen vertex $\rho _n$, say the first one revealed in the process that is on the boundary of $P_n$ but is not in the boundary of *H*. From the domain Markov property, $(M_n,\rho _n)$ has the law of the half plane UIPT, and is independent of $P_n$. This completes the proof, since $\rho _n$ is eventually constant, and so $(M_n,\rho _n)$ converges to $(H_1,\rho )$. Finally, by translation invariance of $H_1$, we can choose a root for $H_1$ as any function of $L_1$ and the law of $H_1$ will not change.

By induction, the same holds for all subsequent layers. $\square$

#### Proposition 3.2

In each layer $L_i$ we have the following.

(i) The blocks are independent. All have the same law, except for the block containing the root edge which is biased by the size of its lower boundary. Given the block containing the root edge, the root edge is distributed uniformly among the edges in its lower boundary.
(ii) The number of edges *B* in the lower boundary of a block (other than the one containing the root edge) satisfies ${\mathbb {E}}(B) = 1$ and ${\mathbb {P}}(B>t) \sim ct^{-3/2}$.
(iii) Conditioned on the lower boundary length of *B*, the component of *H* within the hole is a Boltzmann map of an $(B+2)$-gon with parameter 2 / 27.

We remark that the proof yields the precise distribution of the lower boundary size of a block in terms of the partition function of triangulations, which is explicitly known. We do not need the formula for this distribution.

#### Proof

We prove the statements for the first level $L_1$. By Lemma 3.1 it holds for all other layers, as they have the same law and are independent of each other.

We enumerate the blocks $\{B_i\}_{i \in {\mathbb {Z}}}$ using integers with $B_0$ being the block containing the root edge. Consider a sequence of blocks $(B_i)_{i\in [j,k] \cap {\mathbb {Z}}}$ with $j\le 0\le k$. Suppose $B_i$ has $b_i$ lower boundary edges and $v_i$ internal vertices in its hole. Let $B_0$ also have a marked edge on its lower boundary. We compute the probability that these are consecutive blocks of $L_1$, with the marked edge of $B_0$ being the root edge. By Euler’s identity we find that a block has $2v_i + b_i + 1$ faces. Joining these blocks, the total number of vertices internal to *M* is $V = 1+\sum v_i+1$, including also the upper boundary vertices. Lemma 2.3, with $F=\sum 2v_i+b_i+1$ gives that the probability of these blocks being part of the map is $6^V 9^{-F}$.

In order for these to be blocks in $L_1$, it is also necessary that if we continue to peel along the boundary to the right than no internal vertex (revealed so far) is swallowed, and that when peeling to the left, the first step reveals a new internal vertex, and afterwards no revealed internal vertex is swallowed. These conditions have probability 1 / 3 and 2 / 27 respectively, which are just a constant. Thus the probability of having the blocks $B_i$ is

$$\begin{aligned} C \prod 6^{1+v_i} 9^{-2v_i-b_i-1} = C \prod \frac{2}{3} \left( \frac{2}{27}\right) ^{v_i} 9^{-b_i}. \end{aligned}$$

for some absolute constant *C*. (Careful calculation shows $C=1$; However, we need not worry about the value of *C*, since it is determined by the fact that these probabilities add up to 1, and its value is canceled out in what follows.) This shows that the blocks are independent, that given $b_i$, and that the hole is filled with a Boltzmann triangulation with parameter 2 / 27. Moreover, the probability that $b_i=m$ is proportional to $\sum _n \phi _{n,m} (2/27)^n 9^{-m}$, which decays as $ct^{-5/2}$ via (2.1). Finally, for $B_0$ there is a marked edge on the lower boundary, so the probability of it having $b_0=m$ is proportional to $\sum _n m \phi _{n,m} (2/27)^n 9^{-m}$, i.e. it is a biased by its lower boundary. That the root is distributed uniformly among the vertices in the lower boundary of its block follows from translation invariance. $\square$

We are going to denote by ${\mathbb {B}}$ the law of a block described in Proposition 3.2 and by ${\mathbb {B}}^{\text {bias}}$ the law of the block containing the root (i.e. biased by the size of its lower boundary). Let $D_{\max }$ denote the maximal degree of a vertex in a block. The following shall come as no surprise to the reader familiar with random maps.

#### Lemma 3.3

Then for some $c,C>0$ and all $r \ge 1$ we have ${\mathbb {B}}(D_{\max } > r) \le C e^{-cr}$, and ${\mathbb {B}}^{\text {bias}}(D_{\max } > r) \le C e^{-cr}$.

The proof follows a fairly standard argument, and is not too difficult, however, we have not been able to locate this statement in the literature. The proof is separated into three steps. The first is known lemma about the degree of a boundary vertex in a Boltzmann triangulation.

#### Lemma 3.4

There are constants *c*, *C* such that for any *m*, if *B* is a Boltzmann triangulation of an *m*-gon, rooted at $\rho \in \partial B$, then ${\mathbb {P}}(d_\rho > r) \le C e^{-cr}$.

#### Proof

This follows from the same argument used in [9] to prove the exponential tail of the degree distribution in the UIPT. $\square$

#### Lemma 3.5

There are constants *c*, *C* such that for any *m*, if *B* is a Boltzmann triangulation of an *m*-gon, rooted at $\rho \in \partial B$, and $D_{\max }$ the maximal degree of any internal vertex, then ${\mathbb {P}}(D_{\max } > r) \le C m^2 e^{-cr}$.

#### Proof

We perform a peeling process to reveal *B*, each time peeling at some edge and revealing one face. However, if the revealed face separates the map into two sub-maps, we do not reveal either of them immediately, but proceed to explore one and then the other in some arbitrary order. Thus at time *i* we have revealed *i* faces, and the remainder of *B* is a collection of independent Boltzmann maps of some cycles (unless the process has terminated, in which case there is no complement).

Let ${\mathcal {F}}_i$ be the sigma algebra generated by this peeling process up to the *i*th step. Let $A_i$ be the event that a new vertex is revealed at step *i*, and let $A_{i,r}\subset A_i$ be the event that this vertex has degree greater than *r*. Our goal is to bound ${\mathbb {P}}(\cup _i A_{i,r})$:

$$\begin{aligned} {\mathbb {P}}(D_{\max }> r)&\le \sum _i {\mathbb {P}}(A_{i,r}) \nonumber \\&\le \sum _i {\mathbb {E}}\left[ {\mathbb {P}}(A_{i,r} | {\mathcal {F}}_i) \right] \le \sum _i {\mathbb {E}}\left[ \mathbbm {1}_{A_i} {\mathbb {P}}(d_i>r | {\mathcal {F}}_i) \right] \end{aligned}$$

since $A_i\in {\mathcal {F}}_i$, where $d_i$ is the degree of the vertex revealed at step *i*. When a new vertex is revealed, its degree is 2. Conditioned on ${\mathcal {F}}_i$, the component of vertex *i* is filled with a Boltzmann map, and so by Lemma 3.4, we have ${\mathbb {P}}(d_i>r | {\mathcal {F}}_i) \le C e^{-cr}$. Thus we have

$$\begin{aligned} {\mathbb {P}}(D_{\max } > r)&\le \sum _i {\mathbb {E}}\mathbbm {1}_{A_i} C e^{-cr} \\&= C e^{-cr} {\mathbb {E}}|B| \le C m^2 e^{-cr}, \end{aligned}$$

Where |*B*| is the number of vertices in *B*, which is known to have expectation of order $m^2$ (see [9, Proposition 5.1]). $\square$

#### Proof of Lemma 3.3

We know from the second item in Proposition 3.2 that the probability that the boundary of a Boltzmann map of law ${\mathbb {B}}$ or ${\mathbb {B}}^{\text {bias}}$ is larger than $e^{\varepsilon r}$ is exponentially small. The rest follows from Lemma 3.5 for $\varepsilon =c/3$, with the constant *c* from Lemma 3.5. $\square$

### Full plane extension of *H*

Given the layer decomposition of the half plane UIPT, we now construct a plane triangulation *M* with no boundary which contains *H* as a sub-map. Eventually we will also show that *M* is almost surely recurrent, implying Theorem 1.

To construct *M* start with the half plane UIPT $H = \bigcup _{i\ge 1} L_i$. We add a sequence of layers below the boundary $S_0$, to create a full plane map. For each $i\le 0$, the layer $L_i$ is composed of a doubly infinite sequence of i.i.d. blocks with law ${\mathbb {B}}$, attached to form a layer. Note that there is no size biased block in $L_i$ for $i\le 0$. Thus for $i\le 0$, if $L_i$ is rooted at some vertex on the top boundary, it is translation invariant in law. We then identify the top boundary of $L_i$ with the bottom boundary of $L_{i+1}$ for every $i\le 0$. The full plane map is defined by $M = \bigcup _{i\in {\mathbb {Z}}} L_i$. By translation invariance of the lower layers, the law of the resulting full plane map does not depend on which edge in the top boundary of $L_{i-1}$ is identified with the root of $L_i$. The boundary between $L_{i+1}$ and $L_{i}$ is denoted $S_i$ also for $i<0$. Vertices of $S_i$ for any *i* are also called skeleton vertices. The map *M* is rooted at the root $\rho$ of *H*. Theorem 1 is an immediate consequence of the following.

#### Theorem 3.6

The full plane extension *M* is almost surely recurrent.

Let $S = \bigcup S_i$ be the skeleton of the map $(M,\rho )$. We define a graph on *S*, where two vertices $x,y\in S$ are adjacent if they are both incident to some hole in some layer of *M*. Call this graph $\text {Skel}(M)$. Note that adjacent vertices in any $S_i$ are adjacent in $\text {Skel}(M)$, and that neighbours in $\text {Skel}(M)$ are either both in $S_i$ for some *i* or in $S_i$ and $S_{i+1}$ for some *i*. Note that $\text {Skel}(M)$ does not have a natural map structure, and is not even planar since a large hole gives rise to large cliques in $\text {Skel}(M)$. However, this graph consists of finite cliques, and the intersection graph between the cliques is planar and approximates *M* in some ways. (We do not rely on this, and so do not make this precise here.) The nonempty blocks in *M* correspond naturally to these cliques in $\text {Skel}(M)$ with the vertices in the boundary of a block forming a clique, and so we will refer to these cliques as blocks in $\text {Skel}(M)$. We shall use the notation $\text {Skel}(A)$ to denote the corresponding graph also for various $A \subset M$, which will be the subgraph of $\text {Skel}(M)$ induced by vertices of *A*.

Our immediate goal is to show that $\text {Skel}(M)$ has polynomial volume growth. The hull of a set *A* of vertices in $\text {Skel}(M)$ is defined to be the set together with all finite components of its complement in $\text {Skel}(M)$. Let $B_{sk}(r)$ denote the hull of the ball in $\text {Skel}(M)$, of radius *r* (in the graph metric) around $\rho$.

#### Proposition 3.7

The random variables $r^{-4} | B_{sk}(r) |$ form a tight family.

Indeed, we expect these random variables converge in distribution.

We start with a few additional definitions. For any skeleton vertex $v \in S_i$ we can associate a unique hole in the layer above it ($L_{i+1}$) that contains the edge of $S_i$ to the right of *v*. (We use the edge, since it is possible for a vertex to intersect multiple holes in the layer above it.) This hole is also incident to $S_{i+1}$ at a unique vertex. Call this vertex the parent *p*(*v*) of *v*, and define $p^{(r)}$ to be the *r* fold composition of the operation *p*. Equivalently, the parent of a vertex $v\in S_i$ is the rightmost vertex of $S_{i+1}$ that is adjacent to *v* in $\text {Skel}(M)$.

Using Proposition 3.2 and Lemma 3.1 it is natural to study the maps via certain critical Galton-Watson trees derived from the block decomposition. A similar construction was used by Krikun for the full plane UIPQ in [25, 26], except that the trees there are not Galton-Watson trees. In the layered map, we define a tree as follows. The vertices are the skeleton vertices. The parent of *v* is *p*(*v*). The set of all offspring of a vertex *v* form a tree, which we denote by $T_v$. The following is clear from this discussion and Proposition Proposition 3.2 and Lemma 3.1.

#### Lemma 3.8

For every skeleton vertex $v\in S_0$, the tree $T_v$ is a critical Galton-Watson tree with offspring distribution *Z* satisfying ${\mathbb {P}}(Z>k) \sim ck^{-3/2}$. Moreover, the law of the (infinite) tree rooted at $\rho$ is the fringe of the same Galton-Watson tree.

Note that in particular the sub-tree of offspring of each vertex *v* is a.s. finite, whereas the entire tree is infinite since every vertex has a parent. See Aldous [2] for the theory of fringes of trees. We do not need this theory in full generality, but use the following consequences:

- Every ancestor $p^{(r)}(\rho )$ of $\rho$ has offspring distributed as size biased *Z*.
- The previous ancestor $p^{(r-1)}(\rho )$ is a uniform child of its parent.
- All other children of $p^{(r)}(\rho )$ produce independent Galton-Watson trees of offspring.

While a-priori it is not obvious that our parent definition defines a single tree and not a forest. The connectivity can be deduced from the criticality of the trees by showing that for any two vertices of $x,y \in S_i, p^{(r)}(x) =p^{(r)}(y)$ for *r* large enough. This is straightforward, but we do not need the connectivity for our purposes, so we omit the details.

#### Lemma 3.9

Let ${\widetilde{B}}(2r)$ be the hull of the ball of radius 2*r* around $p^{(r)}(\rho )$ in $\text {Skel}(M{\setminus }H_r)$, then $r^{-4} |\widetilde{B}(2r)|$ are tight.

Note that $M{\setminus }H_r$ is the half plane map consisting of $L_i$ for $i\le r$, and thus we are considering a ball in the skeleton of this map, centred at a boundary vertex.

#### Proof of Proposition 3.7

The ball $B_{sk}(r)$ is contained in layers $L_{-r},\dots ,L_r$, since the $\text {Skel}$-distance from $\rho$ to $p^{(r)}(\rho )$ is *r*, we have that $B_{sk}(r) \subset {\widetilde{B}}(2r)$. The claim now follows from Lemma 3.9. $\square$

For the proof of Lemma 3.9, we require the following standard estimate regarding survival probabilities for Galton-Watson trees. Note that the offspring distribution in $T_v$ has infinite second moment, so the probability of surviving to level *n* does not decay as *c* / *n* and the volume up to level *n*, conditioned on survival to that level is not quadratic. Recall that it is possible to obtain an infinite version of a critical Galton-Watson tree by conditioning it to survive up to generation *n* and then taking the limit in the local weak topology as $n\rightarrow \infty$. Moreover such an infinite tree has a single infinite path – the spine – and finite trees attached to it. The tree can be described as follows. The root has an offspring distribution which is the size biased version of the original offspring distribution. A uniformly picked child *v* has a tree conditioned to survive below it, while all its siblings have unconditioned Galton-Watson trees of descendants. We refer to [23] for a detailed account of Galton-Watson trees conditioned to survive. The two following lemmas are standard. Proofs can be found e.g. in [16].

#### Lemma 3.10

Let *T* be a critical Galton-Watson tree with offspring distribution satisfying ${\mathbb {P}}(Z>k) \sim c k^{-3/2}$. Then the probability that *T* survives to generation *n* decays as $cn^{-2}$ for some *c*.

#### Lemma 3.11

Let $T^*$ be a critical Galton-Watson tree with offspring distribution satisfying ${\mathbb {P}}(Z>k) \sim c k^{-3/2}$ conditioned to survive. Let $W_n$ be the number of offspring in the *n*th generation of $T^*$, and let $Y_n = \sum _{t=1}^n W_n$. Then $n^{-2}W_n$ and $n^{-3}Y_n$ converge in distribution to some non-zero random variables.

#### Proof of Lemma 3.9

We will construct inductively a growing sequence of subgraphs $$\{P_i\}$$ around $$p^{(r)}(\rho )$$ in such a way that $$P_{i} $$ contains the hull of the ball of radius *i* around $$p^{(r)}(\rho )$$ in $$\text {Skel}(M{\setminus }H_r)$$. For all *i*, all vertices of $$P_i$$ will be in layers $$S_{r-i},\dots ,S_r$$ (as is the ball that they bound). Also it will be clear from the construction that the portions of $$S_i$$ in $$P_r$$ consists of connected segments. This will be useful later in Lemma 4.3.

As a base case, we set $P_0 = \{p^{(r)}(\rho )\}$. We also define a two-sided sequence of vertices in $S_r$, starting with $U_0 = \{p^{(r)}(\rho )\}$. For $i>0$, having defined $P_{i-1}$, and $U_{1-i},\dots ,U_{i-1}$, let $U_i$ be the nearest vertex in $S_r$ to the right of $U_{i-1}$ such that the tree below $U_i$ survives for at least *i* generations. Similarly, $U_{-i}$ is the nearest vertex in $S_r$ to the left of $U_{1-i}$ such that the tree below $U_{-i}$ survives for at least *i* generations.

**Fig. 3:**
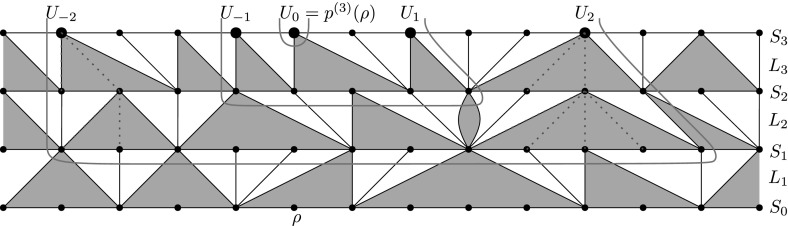
Construction of the maps $P_0$,$P_1$,$P_2$ in the proof of Lemma 3.9. The vertices $U_{-2},\dots ,U_2$ are larger. The first two generations of the trees below $U_{\pm 2}$ are *dotted in blue*. The boundaries of the maps are *marked in red*. Each boundary forms a cutset around the previous one (colour figure online)

We now define $P_i$ as follows. We take all vertices in the first *i* generations of the trees below $U_{-i}$ and $U_i$ (i.e. from $S_r$ down to $S_{r-i}$). Since the definition of the trees is asymmetric in that of the holes, it is convenient for the tree at $U_i$ to also take the rightmost vertex of the rightmost hole visited at each level. (This vertex is not in the tree below $U_i$, but just adjacent to the right). Finally, we also take in each of these levels all vertices between these two trees. See Fig. 3 for an illustration.

Note that since the first *i* levels of the tree below $U_i$ are strictly to the right of the tree below $U_i$, and similarly on the left, we have that $P_i$ indeed form an increasing sequence of subgraphs.

The rest of the proof consists of two claims. First, that the set $P_i$ contains the ball of radius *i* around $p^{(r)}(\rho )$ in the map $\text {Skel}(M{\setminus }H_r)$. Secondly, we estimate of the size of these sets.

The first claim is proved by induction. Clearly the claim is true for $P_0$. For the induction step, we argue that the internal boundary of $P_i$ (i.e. vertices of $P_i$ connected to its complement) is completely contained in the two trees below $U_{\pm i}$ together with the segment of $S_{r-i}$ between the two trees. In particular, the boundary is disjoint of $P_{i-1}$, and hence each $P_i$ contains a ball of radius one around $P_{i-1}$.

A level $S_j$ is naturally partitioned into intervals of vertices with a common parent. The lower boundary of a hole is one such interval, together with the first vertex of the next interval to the right. Edges of $\text {Skel}(M)$ are either within intervals, or between adjacent intervals, or between a vertex and its parent, or between a vertex and the parent of an adjacent interval. Since every level from $S_{r-i},\dots ,S_r$ contains some vertices from the trees under $U_i$ and $U_{-i}$ these two trees indeed separate the rest of $P_i$ from vertices to the right and left. Clearly only vertices in $S_{r-i}$ can be connected to vertices further down in the map, and the first claim is proved.

Finally, we consider the size of $P_{2r}$, which consists of the first 2*r* generations from the trees rooted at each vertex between $U_{-2r}$ and $U_{2r}$. The tree rooted at $U_0$ is special: Its first *r* levels are those of the tree conditioned to survive, with one vertex at each level having the size-biased offspring distribution. After level *r*, it transitions to a critical tree. The trees at all other vertices of $S_r$ are critical Galton-Watson trees, except that the choice of $U_i$ is not independent of the trees.

Fix some $\varepsilon >0$. For the tree at $U_0$, by Lemma 3.11 we have for some *C* that the number of vertices in generations $0,\dots ,r$ is at most $Cr^3$ and the number of vertices at generation *r* is at most $Cr^2$ with probability at least $1-\varepsilon$. Below each of the vertices at generation *r* we consider the first *r* generation of an independent critical Galton-Watson tree. On the event that generation *r* is not too large, this adds in expectation at most another $Cr^3$ vertices, and by Markov’s inequality the total contribution from the tree at $U_0$ is at most $(C+C/\varepsilon ) r^3$ with probability at least $1-2\varepsilon$.

The trees at other vertices of $S_r$ are all independent critical Galton-Watson trees, and we consider the first 2*r* levels of these trees. The expected size of each such tree is $2r+1$ (including its root). It is convenient to identify the vertices of $S_r$ with ${\mathbb {Z}}$, with $U_0$ being 0. Between $U_{i-1}$ and $U_i$ we consider trees until finding one that survives to generation *i*, and so $U_i$ is a stopping time. Since $U_i-U_{i-1}$ is geometric with mean of order $Ci^2$ (by Lemma 3.10), we have ${\mathbb {E}}U_{2r} \le Cr^3$. By Wald’s identity, the expected total size of the trees from $U_1$ to $U_{2r}$ is at most $C r^4$. By symmetry, the same holds for trees to the left of $U_0$, and the claimed tightness follows. $\square$

## *M* as a distributional local limit

We now define a sequence of finite maps $M_n \subset H$. Each $M_n$ will inherit the layered structure from *H*, and so some vertices of $M_n$ will be designated skeleton vertices. The maps $M_n$ will have the property that if we select a root $\rho _n$ uniformly from the skeleton vertices, then $(M_n,\rho _n)$ converges in distribution to *M*.

**Fig. 4:**
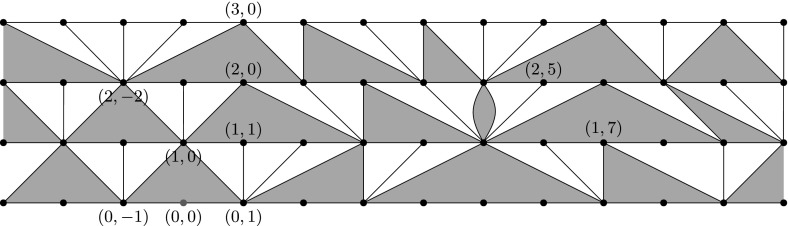
The coordinate system for $\text {Skel}(M)$, with some coordinates noted

For a skeleton vertex $v\in H$, recall the definition of the parent *p*(*v*) of *v* from Sect. 3.2, and that $p^{(k)}$ is the *k*-fold composition of the operation *p*. Now we set up a coordinate system for skeleton vertices as follows. The vertices of $S_k$ will have coordinates $\{(k,n)\}_{n\in {\mathbb {Z}}}$, in the order they occur in $S_k$. The root vertex $\rho$ has coordinates (0, 0) and for any $k>0$, the vertex $p^{(k)}(\rho )$ has coordinates (*k*, 0). Having defined these, the vertex of $S_k$ at a distance *j* to the right (resp. left) of (*k*, 0) has coordinates (*k*, *j*) (resp. $(k,-j)$). See Fig. 4 for an example. Note that coordinates are only defined for vertices in $S_k$ with $k\ge 0$.

This coordinate system has several useful properties. For instance:

### Lemma 4.1

Let $B_{ij}$ denote the block in $L_i$ incident the vertices (*i*, *j*) and $(i,j+1)$ of $S_i$. Then the blocks $B_{ij}$ are independent, the blocks $\{B_{i0}\}_{i \ge 0}$ are have law ${\mathbb {B}}^{\text {bias}}$ and the rest have law ${\mathbb {B}}$. Further, for any $i\ge 0$ and $j\in {\mathbb {Z}}$, conditioned on $L_1, \dots , L_i$, the map $H_{i}$ rooted at (*i*, *j*) has law ${\mathbb {H}}$.

### Proof

The first assertion follows from Proposition 3.2 while the second assertion follows from domain Markov property and translation invariance of *H*. $\square$

### Lemma 4.2

There exist constants $c,c'>0$ such that for all $k \ge 1$ and all $i,j \in {\mathbb {Z}}$, the following holds. Let *v* be the vertex with coordinate (*i*, *j*). Then

$$\begin{aligned} {\mathbb {P}}(\deg (v) >k )\le ce^{-c'k}. \end{aligned}$$

### Proof

Notice that the vertex *v* can have neighbours in only one block in the layer below it and in possibly several blocks in the layer above it. We first show that the number of such blocks has geometric tail. Indeed, one of the blocks in the layer above *v* is a size biased block *b*. If *v* is in the leftmost vertex in the lower boundary of *b*, then we need to find the first block $b'$ to the left of *b* with positive lower boundary size. The blocks between $b'$ and *b* (including $b'$ and *b*) contribute to the degree of *v*. Since blocks are i.i.d.  the number of such blocks has geometric tail. On each block, the contribution to the degree of *v* has exponential tail using Lemma 3.3. A similar argument holds if *v* is the rightmost vertex in the lower boundary of *b*. Thus the degree of *v* is a sum of a geometric number of independent random variables, each with exponential tail, which is easily seen to have an exponential tail. $\square$

### Lemma 4.3

Let $\Delta$ be the maximal degree in *M* of the vertices in $B_{sk}(r)$. There exists $C>0$ such that ${\mathbb {P}}(\Delta > C \log r) \rightarrow 0$ as $r \rightarrow \infty$.

### Proof

We use the notations from the proof of Lemma 3.9. The ball is contained in the set $P_{2r}$ defined there, and this set contains a single connected interval from each path $S_i$. Since $r^{-4} |P_{2r}|$ is tight, and $(i,0) \in P_{2r}$ for $i\in [-r,r]$, with high probability $P_{2r} \subset [-r,r] \times [-r^5,r^5]$. The degree of each skeleton vertex $(i,j)\in [-r,r]\times [-r^5,r^5]$ has an exponential tail by Lemma 4.2 and a union bound completes the proof. $\square$

Another crucial property of the coordinate system is the following.

### Lemma 4.4

Fix $k \ge 0$, and define $\ell ' = \ell '(k,\ell )$ by $(k+1,\ell ') = p((k,\ell ))$. Then almost surely,

$$\begin{aligned} \frac{\ell '}{\ell } \xrightarrow [\ell \rightarrow \infty ]{} 1. \end{aligned}$$

### Proof

The blocks in layer $L_{k+1}$ corresponding to vertices $(k+1,i)$ for $i>0$ form an i.i.d. sequence of blocks distributed as ${\mathcal {B}}$. The statement is now an immediate consequence of Proposition 3.2 (specifically that ${\mathbb {E}}B_i = 1$) and the Strong Law of Large Numbers which implies $\ell /\ell ' \rightarrow 1$. $\square$

We define $M_n$ as follows. The skeleton vertices of $M_n$, denoted $\text {Skel}(M_n)$ is the set $\{(i,j): 0 \le i< n,0 \le j < n\}$. The holes of $M_n$ includes all holes in *H* all of whose skeleton vertices are contained in $\text {Skel}(M_n)$. The edges of $M_n$ are those edges of *H* both of whose vertices belong either to the above described skeleton vertices or to one of the above described holes. Finally take a root $\rho _n$ for $M_n$, which is a uniformly selected selected skeleton vertex from $M_n$.

The following lemma is a corollary of Lemma 4.2.

### Lemma 4.5

There exist constants $c,c'>0$ such that for all $n \ge 1$,

$$\begin{aligned} {\mathbb {P}}(\deg (\rho _n) >k) \le ce^{-c'k}. \end{aligned}$$

Next, we show that $\rho _n$ is far from the ‘boundary’ of $M_n$ with high probability.

### Lemma 4.6

We have the convergence in distribution

$$\begin{aligned} d_\text {Skel}(\rho _n, \partial M_n) \xrightarrow [n\rightarrow \infty ]{(d)} \infty , \end{aligned}$$

where $d_\text {Skel}$ is graph distance in $\text {Skel}(M_n)$.

### Proof

Consider the graph on the skeleton vertices, with an edge (*x*, *y*) if they are either at the same level and adjacent, or one is the parent of the other. Let ${\tilde{d}}$ be the distance in this graph. It is easy to see that ${\tilde{d}}(x,y) \le 3d_\text {Skel}(x,y)$, so it suffices to prove that for arbitrary *r*, with high probability ${\tilde{d}}(\rho _n, \partial M_n) \ge r$. Let ${\tilde{B}}$ denote the metric balls of ${\tilde{d}}$.

Observe that $M_n$ has $n^2$ skeleton vertices by definition. Let the root $\rho _n$ have coordinates (*i*, *j*). Clearly, *i*, *j*, and $M_n$ are all independent. As $n\rightarrow \infty$, with high probability $r< i < n-r$. On this event, the ${\tilde{d}}$-ball ${\tilde{B}}(\rho _n,r)$ does not intersect $S_0$ or $S_n$. Fix any such *i*. By Lemma 4.4, for any $\varepsilon >0$ there is some *M* so that with probability at least $1-\varepsilon$ the following holds: For every vertex $(x,y) \in [i-r,i+r] \times [M,n)$, if $(x',y')$ is ${\tilde{d}}$-adjacent to (*x*, *y*), then $y'/y \in (e^{-\varepsilon },e^{\varepsilon })$. Call this event $B_i$, and assume it holds. If $\rho _n=(i,j)$ and $j>e^{r\varepsilon } M$ then every vertex in ${\tilde{B}}(\rho _n,r)$ has second coordinate in $[e^{-r\varepsilon } j, e^{r\varepsilon }j]$. If *n* is large enough, then with high probability $e^{r\varepsilon } M< j < e^{-r\varepsilon } n$, and then the ball ${\tilde{B}}(\rho _n,r)$ is contained in $M_n$. $\square$

### Proposition 4.7

We have the limit $(M,\rho ) = \lim (M_n,\rho _n)$ in the weak local topology.

### Proof

The coordinates $(i_n,j_n)$ of a the uniform root $\rho _n$ tend to infinity in distribution as $n\rightarrow \infty$. By Lemma 4.6 large balls around $\rho _n$ are contained in $M_n$, so the weak local limit of $(M_n,\rho _n)$ is the same as the limit of $(M,\rho _n)$. Since $i_n,j_n$ are independent of *M*, it suffices to show that for a fixed sequence $\{(i_n,j_n)\}\rightarrow \{\infty ,\infty \}$, if we take $\rho _n = (i_n,j_n)$, then $(M,\rho _n)$ converges in distribution to the full plane map $(M,\rho )$.

Given the layers $L_1,\dots ,L_i$, the half plane map above $L_i$ (namely, $H_i$) has law ${\mathbb {H}}$. Thus the layers above $\rho _n$ have the law of the HUIPT, and are independent of the layers below $\rho _n$. Note that translation invariance implies that the block above $\rho _n$ is precisely size-biased, as are subsequent blocks above it.

Since the blocks in the first *i* layers are independent with law ${\mathbb {B}}$, the layer below $\rho _n$ has law equal to that of layer $L_0$ in *M*, except for one block at distance $j_n\rightarrow \infty$. The same is true for all levels below $\rho _n$. By Lemma 4.6, the distance to these biased blocks tends to infinity. Thus layers below $\rho _n$ in $\lim (M,\rho _n)$ have i.i.d. blocks, which is the law of these layers in *M*. $\square$

## Bounding degrees: the star-tree transformation

Following [20], we apply the so-called star-tree transformation to our maps to get maps with bounded degrees. These can then be embedded in the plane using circle packings, which are better behaved when vertices have bounded degrees.

The star-tree transform is constructed roughly as follows: starting with a map *G*, possibly with large degrees, take its dual, which may have faces of large degree. Triangulate each face to get a triangulation, and take the dual again to get a three regular map $G'$ which is related to the original map. The triangulation step can be done in various ways, and we will be more specific below. The complete process has the effect of replacing each vertex of *G* by a 3-regular tree which connects to other trees at its leaves. Crucially for the recurrence arguments, we make all of these trees as balanced as possible, so that a vertex of degree *d* (star) is replaced by a tree of diameter $O(\log d)$.

**Fig. 5:**
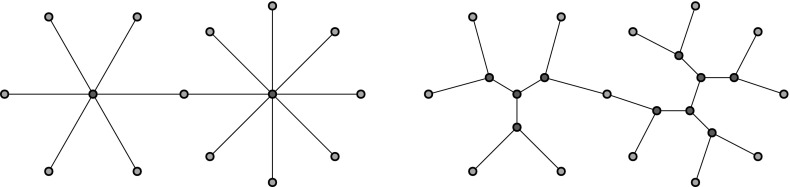
The star tree transform. *Blue* nodes are vertices of *G*, while *yellow* nodes are the vertices created when cutting edges in half. Here, vertices of degree 6 and 8 are replaced by balanced binary trees with the same number of leaves (colour figure online)

To make this precise, we first cut every edge in half, so that every vertex becomes a star with *d* leaves. Next, each such star is replaced by a balanced tree with $d-2$ internal vertices of degree 3 and *d* leaves. The leaves are in bijection with the leaves of the star that the tree is replacing, in cyclic order. The leaves are identified as in the original map with leaves on other trees. This creates a map with maximal degree 3. (The new map is not 3-regular, since vertices of degree 1 or 2 maintain their degree and identified leaves have degree 2.) The choices of tree for each vertex is arbitrary, except for being maximally balanced. See Fig. 5 for an illustration.

When the star-tree transform is applied to a map *G*, we call the resulting map $G'$. Clearly *G* is a minor of $G'$, as it can be recovered by contracting each tree back to a single vertex. A vertex of degree *d* in *G* corresponds to $(d-2)\vee 1$ vertices in $G'$. Edges in the map $G'$ are now assigned conductances. All edges of a tree associated with a vertex of degree *d* are given conductance $w_e=d$. With these notations, we have the following lemma.

### Lemma 5.1

[20] Let *G* be a planar map, and $G'$ the weighted star-tree transform of *G*. If $G'$ is recurrent, then so is *G*.

For a rooted map, we can give the transformed map $G'$ a root $\rho '$ by choosing uniformly a root within the tree (including the leaves) corresponding to $\rho$.

Recall the rooted graph $(M_n,\rho _n)$ from Sect. 4, where some vertices are designated skeleton vertices. Apply the star-tree transform to $M_n$ to get a finite map $M'_n$. A vertices of $M'_n$ is said to be a skeleton vertex, if it is in the tree associated with some skeleton vertex of $M_n$, including the leaves of that tree.

We consider two ways to choose a root for $M'_n$. First, we could choose a root uniformly among all skeleton vertices of $M'_n$. The law of the resulting rooted map is denoted $\nu _n$. We take an arbitrary subsequential limit of $\nu _n$ and call it $\nu$. A differnet choice is to take the rooted map $(M_n,\rho _n)$, and take the root of $M'_n$ to be a uniform vertex from the tree associated with $\rho _n$. We call the law of this rooted map $\mu _n$. Note that the star tree transform is continuous in the local topology. Since $(M_n,\rho _n)$ converges to $(M,\rho )$ we have that $(M'_n,\rho '_n)$ converges to $(M',\rho ')$, with law $\mu =\lim \mu _n$, where $\rho '$ is a uniform vertex in the tree associated to the root $\rho$ of *M*.

### Lemma 5.2

The measure $\mu$ is absolutely continuous with respect to $\nu$.

### Proof

Given $M_n$, each skeleton vertex of $M_n'$ is equally likely to be the root under $\nu _n$. Under $\mu _n$ a skeleton vertex in the tree of a vertex $v\in M_n$ has probability proportional to $(2\deg (v)-2)^{-1}$ of being the root, since we need to choose $\rho _n=v$ and the associated tree has $2\deg (v)-2$ vertices. Thus $d\mu _n/d\nu _n$ is $C_n (2\deg (\rho _n)-2)^{-1}$ for some $C_n$ in case the root $\rho _n'$ is an interior vertex (i.e. not a leaf) of the tree corresponding to $\rho _n$. Since every skeleton vertex has degree at least $2, d\mu _n/d\nu _n \le C_n/2$ in this case. A small correction is needed in the case the root is an identified leaf between skeleton vertices *u* and *v*. In that case $d\mu _n/d\nu _n$ is at most $C_n (2\deg (u)-2)^{-1} + C_n (2\deg (v)-2)^{-1} \le C_n$. An easy calculation shows that

$$\begin{aligned} C_n(G',\rho ') = \frac{\sum _{v \in V(G) }(2\deg (v) - 2) - E'}{|V(G)|} \le \frac{2\sum _{v \in V(G) }\deg (v) }{|V(G)|} \end{aligned}$$

where *V*(*G*) is the set of skeleton vertices in *G* and $E'$ is the number of edges between skeleton vertices in $G'$ which accounts for the double counting when the leaves of two adjacent skeleton vertices are counted. Clearly $C_n \ge 0$. Let ${\mathbb {E}}^{\nu _n}$ denote the expectation under the measure $\nu _n$. For any event ${\mathcal {E}}$,

$$\begin{aligned} (\mu _n({\mathcal {E}}) )^2\le ({\mathbb {E}}^{\nu _n}(C_n1_{{\mathcal {E}}})/2)^2\le & {} {\mathbb {E}}^{\nu _n}(C_n^2/4)\nu _n({\mathcal {E}}) \le {\mathbb {E}}\Big ( \big ({\mathbb {E}}(\deg (\rho _n) |M_n) \big )^2\Big )\nu _n({\mathcal {E}}) \\\le & {} {\mathbb {E}}\Big ({\mathbb {E}}(\deg ^2(\rho _n) |M_n) \Big )\nu _n({\mathcal {E}}) ={\mathbb {E}}(\deg ^2(\rho _n))\nu _n({\mathcal {E}}) \end{aligned}$$

where in the second inequality we applied Cauchy–Schwarz and in the fourth inequality above, we used Jensen’s inequality. Now we use the fact that $\sup _n {\mathbb {E}}(\deg ^2(\rho _n)) < \infty$ which follows from the exponential tail of the degree of the root of $M_n$ as is proved in Lemma 4.5. This completes the proof as if $\nu ({\mathcal {E}}) =0$, then $\mu ({\mathcal {E}})=0$ by taking limits in the above inequality. $\square$

Finally in order to use circle packing, it is useful to work with triangulations. We triangulate each face of $M_n'$ and of $M'$ to obtain a triangulation. This can be done while maintaining bounded degrees, as in [14]. By a slight abuse of notation, we also denote the resulting maps by $(M_n',\rho _n')$ and $(M',\rho ')$ and their law by $\nu _n$ and $\nu$. Since adding edges cannot turn a transient graph to a recurrent one (by the Rayleigh Monotonicity Principle), we immediately deduce the following using Lemmas 5.1 and 5.2.

### Corollary 5.3

If $(M',\rho ')$ is $\nu$-almost surely recurrent, then $(M,\rho )$ is almost surely recurrent, as is its subgraph *H*.

Finally, we shall also need a simple lemma relating adjacency in $M_n$ and $M'_n$. Let $\pi :M'_n\rightarrow M_n$ be the projection mapping each vertex in the tree corresponding to a vertex *v* to *v*. A vertex arising from the splitting of an edge in two is mapped (arbitrarily) to one of the two endpoints of the edge.

### Lemma 5.4

If $u\sim v$ in $M'_n$, then either $\pi (u)=\pi (v)$ or else $\pi (u)\sim \pi (v)$.

### Proof

Since $M_n$ is a triangulation, after vertices are replaced by trees, each face of $M'_n$ consists of paths from three trees corresponding to a face of $M_n$. These paths are joined at three vertices corresponding to the edges of the face in *M*. All additional edges added to make $M'_n$ into a triangulation connect vertices within a face. $\square$

## Recurrence via circle packing

All the tools are in place, and we are ready to build on the methods of [14, 20] to prove our main result. Throughout this section we have maps $(M_n',\rho _n')$ and $(M',\rho ')$ with law $\nu _n$ and $\nu$ respectively.

Let us recall some useful terminology. Given a finite set of points ${{\mathcal {C}}}$ in a metric space, the radius of isolation $R_x$ of a point $x\in {{\mathcal {C}}}$ is the minimal distance to another point of ${{\mathcal {C}}}$. Following [14], we say that a point $x\in {{\mathcal {C}}}$ is $(\delta ,s)$-unsupported if all but at most *s* of the points in $B(x,\delta ^{-1}R_x)$ can be covered by a ball of radius $\delta R_x$. Otherwise it is $(\delta ,s)$-supported. A key idea in [14], is that for small $\delta$ and large *s*, a finite set cannot have too many $(\delta ,s)$-supported points. We use a quantitative form of this, which appears in [20, Lemma 3.4]:

### Lemma 6.1

[20] There exists some *A*, so that for any finite ${{\mathcal {C}}}\subset {\mathbb {R}}^2$, for all $\delta \in (0,1/2)$ and $s \ge 2$, the fraction of $(\delta ,s)$-supported points in ${{\mathcal {C}}}$ is at most $\frac{A\log (\delta ^{-1})}{\delta ^2 s}$.

In previous work, this lemma was applied to the set of centres of a circle packing of a given graph. A key difference from previous work, is that we take the set ${{\mathcal {C}}}$ to be the set of centres of the circles corresponding to skeleton vertices, and not all vertices. Let $P_n$ be some (arbitrarily chosen) circle packing of $M'_n$ in ${\mathbb {R}}^2$ (which exists in light of the Circle Packing Theorem [24]). Since $M'_n$ is a bounded degree triangulation (with boundary), we may take $P_n$ so that ratios of radii of adjacent circles are bounded. Note that $M_n'$ might not be connected (in fact it is not with constant probability). However, a circle packing of disconnected graph still makes sense. However, the local limit of the skeleton of $(M_n', \rho '_n)$ is indeed connected, which is all we need in what follows.

Having fixed some circle packing for $M'_n$, we now consider the uniform skeleton root $\rho '_n$. Apply a translation and dilation to $P_n$ so that the circle corresponding to the root $\rho '_n$ is the unit disc, and let *Q* be the image of ${{\mathcal {C}}}$ after this transformation, which is now defined on the same probability space as $H, M_n$ and $M'_n$. We have the following easy consequence of Lemma 6.1

### Lemma 6.2

Let $E_r$ be the event that all but at most $r^3$ points of $Q\cap \{|z|<r\}$ can be covered by a disc of radius $r^{-1}$. There exists some *A*, such that for all $r \ge 2, n \ge 1$ we have ${\mathbb {P}}(E_r) > 1-\frac{A\log r}{r}$.

### Proof

Take an arbitrary sample of $M'_n$, and take ${{\mathcal {C}}}$ to be the set of the centres of circles of skeleton points in $M'_n$. For a uniform vertex *v*, scale so that the circle of *v* is the unit circle. By the Ring Lemma, the radius of isolation $R_v$ is in [1, *C*] for some absolute constant *C*.

Now apply Lemma 6.1 with $s=r^3$ and $\delta = 1/(Cr)$. We find that if *v* is uniform in ${{\mathcal {C}}}$, with the claimed high probability, all but at most $r^3$ points in ${{\mathcal {C}}}\cap \{|z|\le Cr R_v\}$ can be covered by a disc of radius $R_v/Cr$. Since $Cr R_v \ge r$ and $R_v/Cr \le 1/r$, this implies the claim. $\square$

Consider the subgraph of $M'_n$ induces by vertices in the disc $\{|z|<r\}$. Let $\Gamma (n,r)$ denote the connected cluster of $\rho '_n$ in this graph, and let ${\bar{\Gamma }}(n,r)$ denote $\Gamma (n,r)$ together with all edges connecting the cluster to vertices outside $\{|z|>r\}$. A major step in our proof is to show that for some constant $\alpha$, the resistance in ${\bar{\Gamma }}(n,r)$ from $\rho '_n$ to the complement of $\{|z|>r\}$ is at least $\alpha$ with high probability. Of course, this is the same as the resistance in $M'_n$ between the same vertex sets. Moreover, we shall prove all this not just for the resistance from $\rho '_n$, but from any finite neighbourhood of $\rho '_n$, i.e. there is some $\alpha >0$ so that for any finite set *A*, the resistance from *A* to the complement of $\{|z|>r\}$ is at least $\alpha$ for *r* large enough. Towards this, we first prove that (with high probability) the maximal conductance of any edge in ${\bar{\Gamma }}(n,r)$ is at most $C\log r$, and that if all conductances are changed to 1 then the resistance between the involved vertex sets is at least $c\log r$. (Recall that the conductance of an edge is the degree of the vertex corresponding to it before the star-tree transform.) The claim then follows by Rayleigh monotonicity with $\alpha =c/C$. In what follows, $R_{\mathrm {eff}}(A,B; w)$ denotes the resistance from *A* to *B* with edge weights *w*. The graph is implicit and should be clear from the context.

### Lemma 6.3

Fix *k*, and let $B_k\subset M'_n$ be the ball of graph radius *k* around $\rho '_n$. For some $c_1$, for all *r* large enough but such that the set of vertices of $M_n'$ outside $\{|z|<r\}$ is nonempty, we have the bound (in ${\bar{\Gamma }}(n,r)$)

$$\begin{aligned} R_{\mathrm {eff}}(B_k,\{|z|\ge r\}; 1) \ge c_1\log r. \end{aligned}$$

### Proof

The radius of the circle of $\rho '_n$ is 1. By the Ring Lemma, radii of adjacent circles have bounded ratio, so every vertex of $B_k$ is contained in $\{|z|<r'\}$ for some $r'=r'(k)$. The resistance across the annulus $\{r'<|z|<r\}$ is now seen to be at least $c\log (r/r')$ by the arguments of [14, 22] (see for example [20, Corollary 3.3]). $\square$

### Lemma 6.4

Let $w_{\max }$ denote the maximal conductance of any edge in ${\bar{\Gamma }}(n,r)$. Then for some $c_0$ we have

$$\begin{aligned} \lim _{r\rightarrow \infty } \limsup _{n\rightarrow \infty } {\mathbb {P}}(w_{\max } \ge c_0 \log r) = 0. \end{aligned}$$

### Proof

Fix $\varepsilon >0$, and consider the event $E_r$ of Lemma 6.2. For $r\ge r_0(\varepsilon )$ we have ${\mathbb {P}}(E_r) \ge 1-\varepsilon$. Assume $r\ge 1$ and that $E_{2r}$ holds, and let $U=\{|z-z_0|<1/2r\}$ be a disc such that $\{|z|<2r\}\setminus U$ contains at most $(2r)^3$ skeleton vertices.

We consider several possibilities according to the location of *U*. If *U* is wholly outside $\{|z|<r\}$, then $\{|z|<r\}$ contains at most $8r^3$ skeleton vertices. Otherwise, $|z_0|<r+1/2$ (since $r\ge 1$). Suppose *U* contains at least 2 vertices, which therefore have circles of radius at most 1 / *r*. Let $U_a = \{|z-z_0| < a/r\}$. From the Ring Lemma it follows that for some *a*, the vertices in the annulus $U_a\setminus U$ disconnect *U* from the complement of $U_a$. In that case, since $M'_n$ is a triangulation, there is a cycle in that annulus that surrounds *U*. For *r* large enough, this cycle lies in $\{|z|<2r\}{\setminus }U$, and so the cycle passes through at most $8r^3$ skeleton vertices (and possibly some non-skeleton vertices).

**Fig. 6:**
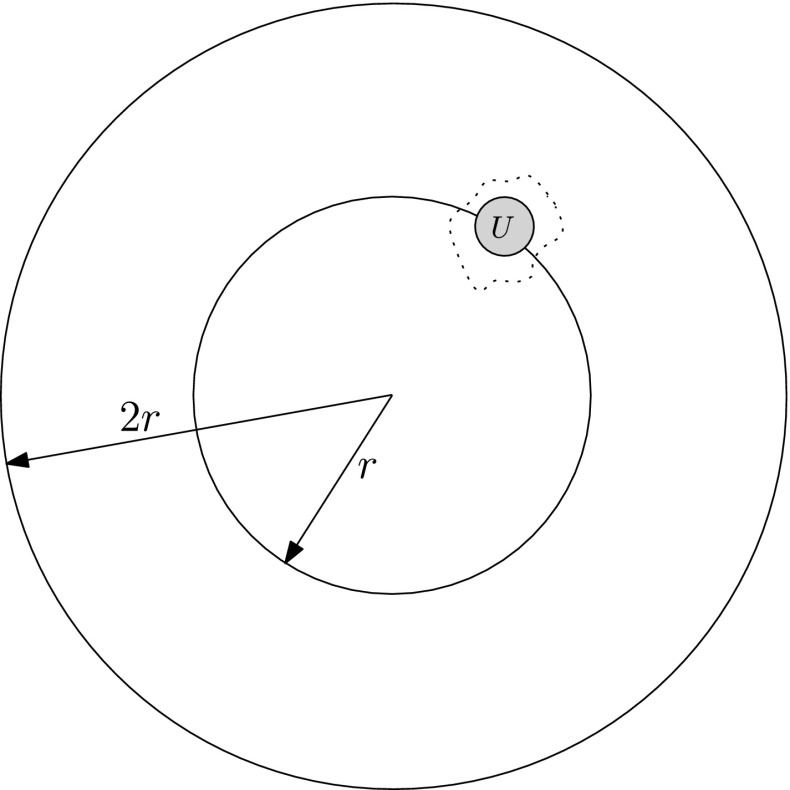
Illustration of the proof of Lemma 6.4. The *grey* disc *U* may include an arbitrary number of skeleton vertices, but the rest of the large disc, including the cycle around *U* contain at most $8r^3$ skeleton vertices. The cycle is used if *U* intersects but is not contained in the disc of radius *r*

Let us summarize our findings so far. For *r* large enough and any *n*, with probability at least $1-\varepsilon$, there is a set $\Lambda$ of at most $8r^3$ skeleton vertices in $M'_n$ that contains every skeleton vertex in $\{|z|<r\}$ except possibly those in *U*. If any vertex from *U* are not in $\Lambda$, the set also contains all skeleton vertices from a cycle separating *U* from $\rho '_n$. That cycle need not be contained in $\{|z|<r\}$. See Fig. 6.

Now, any path $\gamma$ in $M'_n$ which does not contain any boundary vertex of $M_n'$ projects via $\pi$ to a path in $M_n$. The restriction of $\pi (\gamma )$ to the skeleton vertices is a path in $\text {Skel}(M_n)$, which visits no skeleton vertices that are not in $\gamma$ (by Lemma 5.4). For any *r*, for large enough *n* with probability $1-\varepsilon$, no boundary vertex of $M_n'$ is in ${\bar{\Gamma }}(n,r)$ since otherwise, the skeleton distance from $\rho '_n$ to a boundary vertex is at most $8r^3$ (Lemma 4.6). Thus for any *r*, for large enough *n*, with probability $1-2\varepsilon , \Gamma (n,r)$ is contained in the hull of $B_\text {Skel}(\rho '_n,8r^3)$. The result now follows from Lemma 4.3. $\square$

It is clear from the proof of Lemma 6.4 that the set of vertices outside $\{|z|>r\}$ is nonempty with probability tending to 1. Thus, by combining Lemmas 6.3 and 6.4 we get the following with $\alpha =c_1/c_0$.

### Proposition 6.5

Fix an integer *k* and $\varepsilon >0$, and let $B_k\subset M'_n$ be the ball of graph distance *k* around $\rho '_n$. For some $\alpha >0$, for all *r* large enough, we have with probability at least $1-\varepsilon$ as $n\rightarrow \infty$

$$\begin{aligned} R_{\mathrm {eff}}(B_k,\{|z|\ge r\}; w) \ge \alpha . \end{aligned}$$

### Proof of Theorem 1 and Theorem 3.6

The argument is similar to the argument of [20]. We start with the observation that an electrical network *G* is recurrent if and only if for some $\alpha >0$, for every graph distance ball $B_k=B_k(\rho )$ there exists a finite vertex set *S* such that

$$\begin{aligned} R_{\mathrm {eff}}(B_k, G{\setminus }S; w) > \alpha . \end{aligned}$$

Fix *k*, and $\varepsilon >0$. By Proposition 6.5, for any large enough *n*, with probability $1-\varepsilon$ there is some finite *S* such that in $M'_n$ we have $R_{\mathrm {eff}}(B_k, M'_n{\setminus }S; w) > \alpha$. Moreover, with high probability for some *R*, the set *S* is contained in the hull of a ball of radius *R* in $M'_n$. Going to the limit, we find that for *n* large enough, with probability at least $1-2\varepsilon$ the resistance in $M'$ from $B_k$ to the complement of some large finite set *S* is at least $\alpha$. Since $\varepsilon$ is arbitrary, this implies that $M'$ is $\nu$-almost surely recurrent.

By Lemma 5.2, this implies that $M'$ is $\mu$-almost surely recurrent, which in turn also implies recurrence of *M*, and of *H*. $\square$

## Extensions

### Other maps

Keeping in mind possible future applications, we now summarize here the conditions under which our techniques apply. Let $G_n$ be a sequence of finite planar maps and let $S_n \subset G_n$ be a sequence of its sub-maps, called skeleton vertices. Let $\rho _n$ be a vertex selected uniformly from $S_n$. Assume that the triplet $(G_n,S_n,\rho _n)$ satisfy the following assumptions:

(i) $(G_n,S_n, \rho _n)$ converges to $(G,S,\rho )$ in the local topology where *S* is a connected sub-map of *G*.
(ii) The connected components of $G{\setminus }S$ are all finite. The faces of the map induced by *S* are called holes.
(iii) Let $d_{\text {Skel}}$ denote the graph metric on *S* where two skeleton vertices are adjacent if either they are adjacent in *G* or they are both incident to some common hole in *G*. Let $B_{sk}(r)$ denote the map formed by all vertices in *S* within distance *r* from $\rho$ in *G* in $d_{\text {Skel}}$ along with all the finite connected components of its complement. Let $\Delta$ be the maximal degree in *G* of a vertex in $B_{sk}(r)$. Then there exists a $C>0$ such that
  $$\begin{aligned} {\mathbb {P}}(\Delta >C\log r) \xrightarrow []{r \rightarrow \infty } 0. \end{aligned}$$

Our arguments above show that $M_n$ satisfies these conditions: The skeleton is continuous in local topology hence assumption (i) is a consequence of Proposition 4.7. Assumption (ii) holds since the UIHPT is one-ended, and assumption (iii) is clear from Lemma 4.3.

Our proof relies only on these properties of the planar maps, namely we have proved the following:

#### Theorem 7.1

Let $(G_n,S_n,\rho _n)$ be a sequence of planar maps satisfying assumptions (i), (ii) and (iii) and let $(G,\rho )$ be the local weak limit of $(G_n,\rho _n)$. Then *G* is almost surely recurrent.

*Resistance estimates*

From the argument above we also get some explicit estimates on the growth of the resistance in *M*. In the annulus between Euclidean radii $2^n$ and $2^{n+1}$ the maximal degree is of order *n*. Since the resistance across the annulus without weight is at least *C*, this indicates that the resistance to distance $2^n$ is at least $c\log n$, i.e. the resistance to Euclidean distance *R* is $\log \log R$. This argument can be made precise, but we do not pursue this here. It would be interesting to get better bounds on the growth of the resistance (it is believed to grow like $\log$).

*Other classes of maps*

One natural generalization is to consider uniform infinite domain Markov half plane triangulations with self-loops. Such triangulations can be obtained by taking a HUIPT and decomposing every edge into i.i.d. Geometric number of edges and attaching self-loops on one of the vertices in the 2-gons thus formed by tossing a fair coin (see [8] for detailed discussion on this.) Note that a self-loops with any finite triangulation inside it do not effect recurrence or transience so we can delete them. We can now form an equivalent network by collapsing the geometric number of multiple edges into a single edge and giving this edge a conductance which is equal to the number of edges combined to form it. Thus the equivalent network is HUIPT but with i.i.d. geometric conductances on each edge. It can be checked by the diligent reader that our analysis of the HUIPT goes through in this case also, implying recurrence of this case as well.

A more difficult problem is proving recurrence of more general half planar maps. It is easy to see that a layer decomposition is still possible for various other classes of half plane uniform infinite maps. For quadrangulations, a similar layer decomposition was introduced by Krikun in [25]. The main estimate needed is that the maximal degree in the skeleton balls grow logarithmically in the radius (an analogue of Lemma 4.3.) For maps with even larger faces, a layer decomposition is still possible but it becomes more complicated.

*Hyperbolic maps*

A one parameter family of hyperbolic versions of the half plane UIPT were constructed in [8]. A full plane hyperbolic version was constructed in [18] and it was shown in [7] that the half plane versions can be be realized as a sub-map of the full plane ones. One can carry out the layer decomposition and a full plane extension of the half plane maps in almost exactly the same way as done in this paper. We remark that the skeleton of the resulting full plane map is stationary but not reversible. Call such a full plane map $M^{\mathrm{hyp}}$. The volume of the triangulation inside the holes in this situation will have exponential tail. It is not too difficult to see that the lower half of the triangulation in $M^{\mathrm{hyp}}$ is recurrent. In this situation, if we look at the sequence of hulls of radius *r* and uniformly pick a vertex, the map converges locally to some rerooted version of the lower half, and so the maps are a local limit of finite planar graphs with exponential degree distribution. Exploring the connection between $M^{\mathrm{hyp}}$ and the full plane map defined in [18] is also of interest.

*Stationarity*

It is easy to see that if we put appropriate conductances on the edges of $\text {Skel}(M)$ and bias by the degree (in *M*) of the root vertex, we obtain a stationary reversible graph. A similar construction can be carried out for the hyperbolic versions to obtain $\text {Skel}(M^{\mathrm{hyp}})$. For a simple random walk $Y_0,Y_1,\ldots$ in $\text {Skel}(M^{\mathrm{hyp}})$ or $\text {Skel}(M)$, if we let $\ell (Y_i)$ denote the index of the layer below $Y_i$ an application of ergodic theorem lets us conclude

7.1 $$\begin{aligned} \frac{\ell (Y_i)}{i} \rightarrow s \end{aligned}$$

almost surely for some constant *s*. It follows from the results in [7] that $s>0$ almost surely in $\text {Skel}(M^{\mathrm{hyp}})$ and the recurrence result in this paper shows $s=0$ almost surely for $\text {Skel}(M)$. Notice that simple random walk in $M^{\mathrm{hyp}}$ spends a positive fraction of its time in the skeleton vertices (this is easy to see again via stationarity and exponential tail of the volume of the holes). From all this we can deduce the existence of the speed of simple random walk away from the boundary in *H*. This answers a question in [7].

## References

[1] Addario-Berry, L., Reed, B.: Ballot theorems, old and new. In: Horizons of Combinatorics, pp. 9–35. Springer, London (2008)

[2] Aldous D (1991). Asymptotic fringe distributions for general families of random trees. Ann. Appl. Probab..

[3] Angel O (2003). Growth and percolation on the uniform infinite planar triangulation. Geom. Funct. Anal..

[4] Angel, O., Curien, N.: Percolations on random maps I: half-plane models. Ann. Inst. H. Poincaré, (2013). To appear

[5] Angel, O., Hutchcroft, T., Nachmias, A., Ray, G.: Hyperbolic and parabolic unimodular maps. In preparation

[6] Angel O, Hutchcroft T, Nachmias A, Ray G (2016). Unimodular hyperbolic triangulations: circle packing and random walk. Invent. Math..

[7] Angel, O., Nachmias, A., Ray, G.: Random walks on stochastic hyperbolic half planar triangulations. Random Struct. Algorithms (2014). arXiv:1408.4196

[8] Angel O, Ray G (2015). Classification of half planar maps. Ann. Probab..

[9] Angel O, Schramm O (2003). Uniform infinite planar triangulations. Commun. Math. Phys..

[10] Benjamini I, Curien N (2012). Ergodic theory on stationary random graphs. Electron. J. Probab..

[11] Benjamini I, Curien N (2013). Simple random walk on the uniform infinite planar quadrangulation: subdiffusivity via pioneer points. Geom. Funct. Anal..

[12] Benjamini I, Lyons R, Peres Y, Schramm O (1999). Group-invariant percolation on graphs. Geom. Funct. Anal..

[13] Benjamini, I., Lyons, R., Schramm, O.: Unimodular random trees. arXiv:1207.1752 (2012)

[14] Benjamini I, Schramm O (2001). Recurrence of distributional limits of finite planar graphs. Electron. J. Probab..

[15] Caraceni, A., Curien, N.: Self-avoiding walks on the uipq. arXiv preprint arXiv:1609.00245 (2016)

[16] Croydon D, Kumagai T (2008). Random walks on Galton–Watson trees with infinite variance offspring distribution conditioned to survive. Electron. J. Probab.

[17] Curien, N.: A glimpse of the conformal structure of random planar maps. arXiv:1308.1807 (2013)

[18] Curien, N.: Planar stochastic hyperbolic infinite triangulations. arXiv:1401.3297 (2014)

[19] Curien, N., Le Gall, J.-F.: First-passage percolation and local modifications of distances in random triangulations. arXiv:1511.04264

[20] Gurel-Gurevich O, Nachmias A (2013). Recurrence of planar graph limits. Ann. Math. (2).

[21] Gwynne, E., Miller, J.: Convergence of the self-avoiding walk on random quadrangulations to $$\text{SLE}_{8/3}$$ on $$\sqrt{8/3}$$ -Liouville quantum gravity. arXiv preprint arXiv:1608.00956 (2016)

[22] He Z-X, Schramm O (1993). Fixed points, Koebe uniformization and circle packings. Ann. Math. (2).

[23] Kesten H (1986). Subdiffusive behavior of random walk on a random cluster. Ann. Inst. H. Poincaré Probab. Stat..

[24] Koebe, P.: Kontaktprobleme der konformen Abbildung. Hirzel (1936)

[25] Krikun, M.: Local structure of random quadrangulations. arXiv:math/0512304 (2005)

[26] Krikun M (2005). Uniform infinite planar triangulation and related time-reversed critical branching process. J. Math. Sci..

[27] Ménard, L., Nolin, P.: Percolation on uniform infinite planar maps. arXiv:1302.2851 (2013)

[28] Miller, J., Sheffield, S.: An axiomatic characterization of the brownian map. arXiv:1506.03806 (2015)

[29] Watabiki Y (1995). Construction of non-critical string field theory by transfer matrix formalism in dynamical triangulation. Nucl. Phys. B.

